# Comparative analysis of distinct phenotypes in gambling disorder based on gambling preferences

**DOI:** 10.1186/s12888-015-0459-0

**Published:** 2015-04-15

**Authors:** Laura Moragas, Roser Granero, Randy Stinchfield, Fernando Fernández-Aranda, Frida Fröberg, Neus Aymamí, Mónica Gómez-Peña, Ana B Fagundo, Mohammed A Islam, Amparo del Pino-Gutiérrez, Zaida Agüera, Lamprini G Savvidou, Jon Arcelus, Gemma L Witcomb, Sarah Sauchelli, José M Menchón, Susana Jiménez-Murcia

**Affiliations:** 1 Pathological Gambling Unit, Department of Psychiatry, Bellvitge University Hospital-IDIBELL, c/ Feixa Llarga s/n, 08907 L’Hospitalet de Llobregat, Barcelona, Spain; 2 Ciber Fisiopatología Obesidad y Nutrición (CIBEROBN), Instituto de Salud Carlos III (Spain), Barcelona, Spain; 3 Departament de Psicobiologia i Metodologia de les Ciències de la Salut, Universitat Autònoma de Barcelona, Barcelona, Spain; 4 Department of Psychiatry, University of Minnesota Medical School, Minnesota, USA; 5 Department of Clinical Sciences, School of Medicine, University of Barcelona, Barcelona, Spain; 6 Department of Clinical Neuroscience, Karolinska Institutet, Stockholm, Sweden; 7 CIBER Salud Mental (CIBERSAM), Instituto Carlos III, Barcelona, Spain; 8 Departament d’Infermeria de Salut Pública, Salut Mental i Maternoinfantil, Escola Universitària d’Infermeria, Universitat de Barcelona, Barcelona, Spain; 9 Leicester Eating Disorders Service, Leicester General Hospital, Leicester, UK

**Keywords:** Gambling disorder, Subtypes, Gambling preferences, Strategic games

## Abstract

**Background:**

Studies examining gambling preferences have identified the importance of the type of gambling practiced on distinct individual profiles. The objectives were to compare clinical, psychopathological and personality variables between two different groups of individuals with a gambling disorder (strategic and non-strategic gamblers) and to evaluate the statistical prediction capacity of these preferences with respect to the severity of the disorder.

**Method:**

A total sample of 2010 treatment-seeking patients with a gambling disorder participated in this stand-alone study. All were recruited from a single Pathological Gambling Unit in Spain (1709 strategic and 301 non-strategic gamblers). The design of the study was cross-sectional and data were collected at the start of treatment. Data was analysed using logistic regression for binary outcomes and analysis of variance (ANOVA) for quantitative responses.

**Results:**

There were significant differences in several socio-demographic and clinical variables, as well as in personality traits (novelty seeking and cooperativeness). Multiple regression analysis showed harm avoidance and self-directedness were the main predictors of gambling severity and psychopathology, while age at assessment and age of onset of gambling behaviour were predictive of gambling severity. Strategic gambling (as opposed to non-strategic) was significantly associated with clinical outcomes, but the effect size of the relationships was small.

**Conclusions:**

It is possible to identify distinct phenotypes depending on the preference of gambling. While these phenotypes differ in relation to the severity of the gambling disorder, psychopathology and personality traits, they can be useful from a clinical and therapeutic perspective in enabling risk factors to be identified and prevention programs targeting specific individual profiles to be developed.

## Background

Gambling disorder (GD), named before pathological gambling, was classified formally as a mental disorder in 1980 by the American Psychiatric Association [1]. In the 5th edition, the Diagnostic and Statistical Manual of Mental Disorders (DSM) [2] describes it as a maladaptive gambling behavior, persistent and recurrent, which alters the course of one’s personal, family or professional life. Results from different epidemiological studies have identified prevalence rates for GD of between 0.5% and 2.5% in the general population, independent of whether these studies are conducted in Europe [3-5], or the rest of the world [6,7].

Studies examining gambling preferences have identified the important roles of accessibility and availability of gambling, as well as the technical and structural features associated with it. Gamblers have been classified according to whether the rewards associated with their gambling activity are immediate or delayed [8] and/or whether the activity is related to skills or chance [9,10]. Based on this, “active” gamblers are those that engage in activities with immediate rewards (e.g., slot machines, scratch cards, bingo, casinos), while “passive” gamblers are those who engage in activities with more delayed rewards (e.g., lottery) and strategic gamblers (SG), emphasize the importance of individual skills (e.g., cards, poker, craps, sports betting, or stock market investment), while non-strategic gamblers (non-SG), emphasize chance as playing a bigger part (e.g., slot machines and lottery).

There have been several research studies that have investigated sociodemographic variables (such as gender or academic level), psychopathology, personality profiles and gambling preferences [10-13] in gamblers. Odlaug et al. [10] observed that individuals’ preferences for specific types of gambling were associated with certain socio-demographic characteristics. The researchers compared three groups of gamblers who differed in the type of gambling they preferred, such as strategic, non-strategic, or both. The three groups were found to have high rates of depression and substance abuse, although no significant differences between groups were found. Nor were there differences in gambling behaviors, such as money invested, frequency of gambling, or severity of the disorder. However, different gambling preferences were associated with differences in the gamblers’ age and gender profile. Specifically, gamblers over the age of 35 years preferred low skill, high chance gambling activities (non-strategic), whereas gamblers under the age of 35 preferred high skill, low chance gambling (strategic). In addition, non-strategic gamblers were more likely to be female.

However, Stevens and Young [14] and Bonnaire et al. [15], argue that gambling preference is not only explained by socio-demographic and psychopathological factors, but is related to more complex mechanisms, such as emotional and personality factors. The reason as to why individuals engage in gambling activities is also important in determining the type of gambling preference. Research suggests that gamblers seeking excitement prefer skill-based and active gambling, while those using gambling to avoid negative emotions usually choose chance and passive gambling activities [9,16]. In fact, gender differences (with men preferring skill and active gambling and women preferring chance and passive gambling) have also been demonstrated [10] which suggest there may be point out to underlying differences in coping strategies between genders [17]. Consistent with these results, several studies have explored this relationship further. They found that strategic gambling, namely cards or sports betting, is associated with high levels of excitement or arousal in men, while in women it appears to be linked to emotional regulation and avoidance of negative feelings [8,18-20].

However, differences in impulsivity levels between gamblers of strategic vs. non-strategic games have not been consistently reported [21]. Studies focusing on more strategic games have described an association between this type of gambling (namely poker and casino) and severity of the behavior, impulsivity and emotional regulation deficits [22,23], whereas studies looking at non-strategic gambling have found this to be more associated with low sensation-seeking [24].

Due to the high degree of controversy regarding whether specific gambling activities are associated with different profiles (e.g., socio-demographic, psychopathology, personality traits, etc.), and the need to identify potential endophenotypes in GD, this study aims to compare patients who have been grouped according to type of gambling. The aims of the present study are: a) to assess clinical and psychopathological differences among two groups of diagnosed GD patients, differing in gambling preferences (strategic gambling *versus* non strategic); b) to analyze different personality profiles between the two groups; c) to determine how gambling preference predicts severity of the disorder, taking into account socio-demographic factors and psychometric features.

## Method

### Participants

The sample comprised 2010 participants who attended the Pathological Gambling Unit at the University Hospital of Bellvitge Psychiatric Service in Barcelona, Spain, between April 2003 and September 2011. All participants were diagnosed by experienced clinicians, through a semi-structured clinical interview conducted at their first visit. Diagnoses were confirmed using the Diagnostic Questionnaire for Pathological Gambling according to DSM-IV criteria [25], which was administered at the participant’s second visit, along with additional questionnaires.

The majority of participants were men (n = 1819, 90.5%), born in Spain (n = 1850, 92.0%), with an elementary level of education (n = 1131, 56.2%), married or living in partnership (n = 1012, 50.3%) and in employment (n = 1185, 59.0%). The majority mentioned that they were habitual gamblers at arcades with the intention to win a prize (n = 1834, 91.2%). Other pathological gambling that was prevalent included lottery (n = 1739, 86.5%), bingo (n = 1048; 52.1%), cards (n = 776, 38.6%) and casino (n = 456, 22.7%). The less prevalent forms of gambling included stock market (n = 102, 5.1%), Internet (n = 77, 3.8%), and sports betting (n = 51, 2.5%).

Participants were classified into two groups according to the type of gambling activity that they engaged in; 1) non-strategic gamblers (slot-machines, lotteries or bingos; n = 1709; 85.0%), and 2) strategic gamblers (cards, horse races, sports, casino, Internet or stock-market; n = 301; 15.0%).

### Measures

The following instruments were used:

- *Diagnostic questionnaire for Pathological Gambling according to DSM-IV criteria* [25,26]. This questionnaire assesses the 10 criteria for PG according to the DSM-IV [27]. The cutoff is 5. The Spanish version was used as it has been shown to have satisfactory psychometric properties with a high internal consistency (Cronbach’s alpha, α = .95), high overall classification accuracy (hit rate = .95) [26]. The internal consistency in the sample was very good, α = .83.
- *South Oaks Gambling Screen; (SOGS*; [28,29]. This is a 33 item diagnostic questionnaire that measures severity of gambling activity (responses ranging from 0–20) that adequately discriminates between probable PG, problem gamblers, and non-problem players. It is a widely used scale in the early assessment of pathological gambling and has shown good evidence of classification accuracy. The SOGS has demonstrated evidence of reliability, including good temporal stability (test-retest r = .98) and internal consistency (Cronbach’s alpha = .94) [29]. The internal consistency in the sample was excellent, α = .89.
- *Symptom Check List- 90-Revised (SCL-90-R)* [30]. This is a 90-item self-report questionnaire designed to measure current psychopathology and distress level. The questionnaire explores 9 dimensions or psychopathological profiles: somatization, obsession-compulsion, interpersonal sensitivity, depression, anxiety, anger-hostility, phobic anxiety, paranoid ideation, and psychoticism. The test provides three levels of information: overall (GSI: Global Severity Index), dimensional (Positive Symptom Total PST), and discrete symptoms (PSDI: Index Positive Symptom Distress). Spanish validation of this scale [31] showed good internal consistency with Cronbach’s alphas between .81 and .90; and good temporal stability with test-retest correlations between .78 and .90. The internal consistency in the sample was between good (paranoid ideation scale, α = .76) to excellent (global scales, α = .98).
- *Temperament and Character Inventory-Revised (TCI-R*; [32]. This questionnaire consists of 240 items with five-point Likert scale response options. The 7 dimensions of the model of the TCI-R are divided into four for temperament and three for character. The temperamental traits are: Sensation Seeking, Harm Avoidance, Reward Dependence and Persistence. The character traits are: Self-Direction, Cooperation and Self-Transcendence. The internal consistency of the different dimensions of personality in a Spanish sample was good with Cronbach’s alphas between .77 and .84 [33]. The internal consistency in the sample was between good (novelty seeking scale, α = .70) to very good (persistence scale, α = .86).

### Procedure

The Ethics Committee of the University Bellvitge Hospital (CEIC) approved this study. All participants were voluntarily seeking treatment in a specialized unit, which is part of the public health system (Pathological Gambling Unit of the Psychiatry Service at the University Hospital of Bellvitge, in Barcelona, Spain). All of the participants who were approached to take part agreed to participate and a written informed consent was obtained from all. Participation in the study involved two visits. At the first visit, participants took part in a semi-structured, face-to-face interview with a clinician in order to further explore their gambling, psychopathology and personality traits [34]. Socio-demographic data (e.g., education, occupation, marital status) and required additional clinical information was also collected at this time. At the second visit, participants completed all the study questionnaires.

The treatment offered at the Unit is based on the CBT approach and consists of 16 weekly sessions (45 minutes each), the structure and format of which is adapted to the individual patient’s needs. The main objective of the treatment is to teach patients to use CBT strategies in order to resist the urge to gamble and ultimately achieve complete abstinence from gambling behaviors. The topics addressed during the sessions include psychoeducation on the disorder, stimulus control (e.g., avoidance of risk situations), response prevention (alternative and compensatory behaviors), cognitive restructuring, reinforcement and self-reinforcement, social skills training, and relapse prevention techniques. Additionally, data about adherence, relapses, and achievement of intersession tasks is systematically collected during every treatment session. For a more detail description of the treatment offered, please see [34,35].

### Statistical analysis

Statistical analysis was carried out with SPSS 20. Data was analysed using logistic regression for binary outcomes and analysis of variance (ANOVA) for quantitative responses. Due to the large sample size and the high power of statistical comparisons, the effect size was valued through Cohen’s *d* coefficient (considering null size for *d* < 0.20, small size for 0.20 < *d* < 0.50, medium for 0.50 < *d* < 0.80 and large for *d* > 0.80). The relative contribution of the gambling preference on the level of psychopathology (SCL-90-R scores) and the severity of GD (SOGS-total score) was measured with multiple linear regressions, clustering the variables into three blocks. The ENTER procedure used to add each block; the first block included sex, age of onset, studies level and civil status, the second block added the seven TCI-R scores and the third block added the type of game (0=Non-SG vs 1=SG). The incremental predictive validity of each block was determined by the change in the R^2^ coefficient (ΔR^2^). The R^2^ (coefficient of determination) represents the extent to which the outcome is predicted by the independent variables included in each regression; that is, it measures how well the regression line fits the real data.

## Results

### Socio-demographic and clinical comparison between GD patients based on gambling preference

There was a statistically significant difference between both groups for certain socio-demographic characteristics, with the SG group having a higher proportion of males (*p* = .024), of younger age (*p* = .002), born outside Spain (*p* = .041), with higher level of education (*p* < .001), and earlier age of onset (*p* = .004) (Table 1). The statistical significance for these measures, however, must be interpreted with caution due the large sample size and the high statistical power: Cohen’s-d effect sizes were small (lower than 0.50) for all the sociodemographic variables.

**Table 1:** **Socio-demographics**

		Non-SG	SG	^*1*^ *p*	Cohen’s
		(n=1,709)	(n=301)		| *d* |
Gender; *%*	*Males*	89.9%	94.0%	.024	0.151
Age (years); *mean (SD)*		42.5 (13.3)	40.0 (13.0)	.002	0.190
Age of onset (years); *mean (SD)*		36.6 (13.3)	34.1 (12.4)	.004	0.194
Origin; *%*	*Spain*	92.8%	89.4%	.041	0.120
Education level; *%*	*Less than primary*	3.1%	2.0%	<.001	0.277
	*Primary*	57.8%	36.7%		
	*Secondary*	35.5%	47.6%		
	*University*	3.7%	13.6%		
Civil status; *%*	*Single*	33.9%	39.7%	.062	0.105
	*Married - in couple*	52.3%	44.9%		
	*Divorced – separated*	13.8%	15.4%		
Employment status; *%*	*Employed*	59.1%	62.5%	.257	0.07

SD: standard deviation. Non-SG: Non-strategic gambling. SG: Strategic gambling.

^1^*p* calculated with chi-square test for categorical demographics and ANOVA for quantitative variables.

Table 2 reports the logistic regressions and ANOVA outcomes comparing Non-SG and SG groups. Results indicate that the SG group had significantly less smokers and alcohol users, but a higher degree of severity of gambling: higher means in the SOGS-total score, the DSM-IV [28,26] total criteria, the number of problematic forms of games and the debts associated to gambling. The SG group was also suffering from significantly poorer psychological state, indicated by higher means on the SCL-90-R scales: obsessive-compulsive, depressive, anxiety, hostility, psychotic and GSI. No differences emerged between the non-SG and SG group in whether they had previous sought help for their gambling, (0.87 vs 0.76; *p* = .657, *d* = 0.03), in the prevalence of lifetime mental disorders (42.5% vs 45.9%; *p* = .548, *d* = 0.04), in the presence of comorbid mental disorders associated to gambling (33.8% vs 32.0%; *p* = .548, *d* = 0.04) or in the presence of autolysis ideation (21.2% vs 24.4%; *p* = .282, *d* = 0.07) or behaviours (8.0 vs 7.6%; *p* = .833, *d* = 0.01) related to gambling. Cohen’s-*d* coefficients suggest moderate to high effect sizes were achieved for the number of addictive gamblers, maximum bets and amount of money spent (SOGS-item 2).

**Table 2:** **Clinical comparison between GD patients according to the gambling preference**

	Means and prevalence		^ 1 ^ Means or proportions comparisons				
	Non-SG	SG	ϕ= mean difference or odds ratio; d=Cohen’s-d				
	(n=1,709)	(n=301)	p	ϕ	95% CI (ϕ)		d
Smoker (yes); %	75.5%	61.2%	**<.001**	**0.51** ^**✝**^	**0.39;**	**0.67** ^**✝**^	0.311
Number of cigarettes/day; *mean*	22.8	21.4	.148	1.39	−0.49;	3.27	0.110
Alcohol abuse (yes); %	15.5%	9.3%	**.007**	**0.56** ^**✝**^	**0.37;**	**0.85** ^**✝**^	0.187
Other drugs abuse (yes); %	10.0%	9.3%	.702	0.92	0.60;	1.41	0.025
Other addictive behaviours (yes); %	7.8%	11.1%	.061	1.48	0.98;	2.23	0.113
Maximum bets (euros); *mean*	568.2	2938.5	**<.001**	**−2370.3** ^**✝**^	**−2665.5;**	**−2075.1** ^**✝**^	0.603*
Mean bets (euros); *mean*	112.4	572.2	**<.001**	**−459.8** ^**✝**^	**−575.4;**	**−344.1** ^**✝**^	0.337
Cumulate debts (euros); *mean*	7020.9	24327.7	**<.001**	**−17306.8** ^**✝**^	**−21163.1;**	**−13450.5** ^**✝**^	0.397
Number of problematic forms of games ; *mean*	1.14	2.07	**<.001**	**−0.93** ^**✝**^	**−1.00;**	**−0.86**	1.059**
SOGS: Total score; *mean*	9.97	10.91	**<.001**	**−0.94** ^**✝**^	**−1.33;**	**−0.54** ^**✝**^	0.287
DSM-IV. Total criteria; *mean*	6.92	7.38	**<.001**	**−0.46** ^**✝**^	**−0.71;**	**−0.20** ^**✝**^	0.219
SCL-90-R: Somatization; *mean*	0.92	0.99	.215	−0.06	−0.17;	0.04	0.078
SCL-90-R: Obsessive/compulsive; *mean*	1.09	1.20	**.039**	**−0.11** ^**✝**^	**−0.21;**	**−0.01** ^**✝**^	0.130
SCL-90-R: Interpersonal sensitivity; *mean*	1.00	1.06	.237	−0.06	−0.17;	0.04	0.075
SCL-90-R: Depressive; *mean*	1.42	1.57	**.010**	**−0.15** ^**✝**^	**−0.27;**	**−0.04** ^**✝**^	0.162
SCL-90-R: Anxiety; *mean*	0.96	1.09	**.013**	**−0.13** ^**✝**^	**−0.23;**	**−0.03** ^**✝**^	0.155
SCL-90-R: Hostility; *mean*	0.86	0.99	**.016**	**−0.13** ^**✝**^	**−0.23;**	**−0.02** ^**✝**^	0.151
SCL-90-R: Phobic anxiety; *mean*	0.47	0.53	.155	−0.06	−0.15;	0.02	0.087
SCL-90-R: Paranoid Ideation; *mean*	0.86	0.91	.352	−0.05	−0.14;	0.05	0.058
SCL-90-R: Psychotic; *mean*	0.85	0.95	**.034**	**−0.10** ^**✝**^	**−0.20;**	**−0.01** ^**✝**^	0.132
SCL-90-R: GSI score; *mean*	1.00	1.11	**.019**	**−0.11** ^**✝**^	**−0.19;**	**−0.02** ^**✝**^	0.146

Non-SG: Non-strategic gambling. SG: Strategic gambling.

^1^Logistic regression for categorical pathological measures and ANOVA for quantitative outcomes.

^✝^Bold: significant contrast. *Bold: medium effect size (0.5≤d≤0.8). **Bold: large effect size (d≥0.80).

DSM-IV: Diagnostic questionnaire for Pathological Gambling according to DSM-IV criteria. SOGS: South Oaks Gambling Screen.

SCL-90-R: Symptom Check List- 90-Revised.

Table 3 shows the results of the ANOVA comparing personality traits (TCI-R scores) between Non-SG and SG groups. Of the TCI-R scales, only novelty seeking (higher mean score for SG) and cooperativeness (lower mean for SG) were significantly different between groups. The effect sizes of the differences were, however small.

**Table 3:** **Personality scores comparisons between GD patients according to the gambling preference**

	Means		Means comparisons through ANOVA				
	Non-SG	SG	Mean difference (MD) and effect size (Cohens’-d)				
	(n=1,709)	(n=301)	p	MD	95% CI (ϕ)		d
TCI-R: Novelty seeking	108.46	111.44	**.002**	**−2.98** ^✝^	**−4.83;**	**-.13** ^✝^	0.208
TCI-R: Harm avoidance	101.45	100.41	.356	1.04	−1.17;	3.24	0.061
TCI-R: Reward dependence	99.82	100.38	.589	−0.56	−2.57;	1.46	0.036
TCI-R: Persistence	110.45	109.68	.571	0.77	−1.91;	3.46	0.037
TCI-R: Self-directedness	127.58	126.31	.370	1.27	−1.51;	4.05	0.059
TCI-R: Cooperativeness	132.74	130.41	**.045**	**2.33** ^✝^	**0.05;**	**4.61** ^✝^	0.133
TCI-R: Self-Transcendence	64.88	64.18	.490	0.70	−1.29;	2.68	0.045

Non-SG: Non-strategic gambling. SG: Strategic gambling. TCI-R: Temperament and Character Inventory-Revised.

^✝^Bold: significant contrast.

### Contribution of gambling preference on psychopathology and severity of gambling

Table 4 shows the multiple regressions valuing the relative contribution of patients’ sociodemographic variables (sex, age, age of onset, studies level and civil status), personality profile and type of gambling on SCL-90-R depression, anxiety and Global Severity Index (GSI) scale and on the SOGS total score. For each block, the change in the R^2^ coefficients (ΔR^2^ valued the increase of the model prediction due to the set of variables included in that block) and the Beta-coefficients after the incorporation of incomes have been included. A fourth step/block was considered including the interaction of sex and gambler type, but it was rejected due the lack of a significant moderation effect (*p* > .05 for all the outcomes considered in the study). The results of the third block analysis for sex indicated that being male was a significant predictor of lower psychopathology (lower SCL-90-R scores). Older age was associated with higher gambling severity (higher SOGS-total scores) and higher scores on the SCL-90-R depression and PST scales. Low education level was associated with lower SCL-90-R depression scores and being single predicted lower gambling severity. TCI-R scores showed that higher scores in novelty seeking was a significant predictor of higher scores in all of the outcomes, as well as higher scores in harm avoidance (except for SOGS-total), persistence (except for SOGS-total), and self-transcendence (except for SOGS-total). Higher scores in self-directedness was predictive of lower scores in both gambling severity and psychopathology, while high scores in cooperativeness did not affect the level of gambling severity but were significantly predictive of lower scores on the SCL-90-R anxiety, GSI and PST scales. Reward dependence was the only significant predictor of the SCL-90-R PSDI scales (negative association: the higher the TCI-R score, the lower the SCL-90-R score). Finally, regarding the type of gambling, participants who reported a preference for strategic games had higher levels of gambling severity and higher levels of psychopathology (higher SCL-90-R scores). Considering the changes in R^2^ coefficients (ΔR) for each regression-block and the Beta-coefficients sizes, the most relevant predictors of the outcome considered in this study were the personality traits measured with the TCI-R (particularly harm avoidance and self directedness, followed by novelty seeking and self-transcendence). Patient’s age and age of onset were also found to be predictors of gambling severity (SOGS-total), but their predictive value was lower for the levels of psychopathology as per SCL-90-R. The highest GD and psychopathological scores were associated with greater education level, being married or living in couple, and preference for strategic games, but the effect size of the relationships was small.

**Table 4:** **Relative contribution of gambling preference on psychopathology and severity of gambling**

	Outcomes→	SOGS	SCL-90-R				
↓ Predictors		Total	Depression	Anxiety	GSI	PST	PSDI
*1st block*							
Sex: male		-.056*	-.214*	-.214*	-.208*	-.138*	-.195*
Age (years-old)		.290*	.234*	.177*	.215*	.267*	.124*
Age of onset (years-old)		-.512*	-.206*	-.193*	-.211*	-.238*	-.109*
Studies (primary or less)		-.026	-.004	.042	.042	.027	.046
Civil status (single)		-.049	.055*	.040	.066*	.085*	.024
	*Block summary: R* ^*2*^	***.078*** ^***✝***^	***.061*** ^***✝***^	***.056*** ^***✝***^	***.059*** ^***✝***^	***.041*** ^***✝***^	***.044*** ^***✝***^
*2nd block*							
Sex: male		-.005	-.117*	-.126*	-.118*	-.045*	-.129*
Age (years-old)		.235*	.097*	.041	.065	.105*	.031
Age of onset (years-old)		-.372*	-.068	-.065	-.070	-.102*	.009
Studies (primary or less)		-.036	-.056*	-.016	-.020	-.039*	.011
Civil status (single)		-.074*	-.022	-.034	-.016	-.003	-.026
TCI-R: Novelty seeking		.286*	.144*	.147*	.130*	.093*	.175*
TCI-R: Harm avoidance		.050	.294*	.326*	.308*	.311*	.198*
TCI-R: Reward dependence		.043	-.041	.011	-.031	-.010	-.062*
TCI-R: Persistence		.047	.080*	.097*	.098*	.066*	.101*
TCI-R: Self-directedness		-.224*	-.346*	-.223*	-.292*	-.296*	-.234*
TCI-R: Cooperativeness		-.053	.032	-.100*	-.094*	-.110*	-.003
TCI-R: Self-Transcendence		.013	.119*	.185*	.193*	.213*	.106*
	*Block summary: ΔR* ^*2*^	***.184*** ^***✝***^	***.319*** ^***✝***^	***.312*** ^***✝***^	***.372*** ^***✝***^	***.380*** ^***✝***^	***.197*** ^***✝***^
*3rd block*							
Sex: male		-.009	-.120*	-.129*	-.121*	-.047*	-.132*
Age (years-old)		.238*	.099*	.043	.066	.106*	.033
Age of onset (years-old)		-.373*	-.070	-.066	-.072	-.103*	.008
Studies (primary or less)		-.022	-.045*	-.006	-.011	-.033	.019
Civil status (single)		-.079*	-.026	-.038	-.019	-.005	-.028
TCI-R: Novelty seeking		.283*	.141*	.145*	.128*	.092*	.173*
TCI-R: Harm avoidance		.052	.295*	.328*	.309*	.312*	.199*
TCI-R: Reward dependence		.039	-.043	.008	-.033	-.012	-.064*
TCI-R: Persistence		.049	.082*	.098*	.099*	.067*	.102*
TCI-R: Self-directedness		-.223*	-.346*	-.222*	-.291*	-.296*	-.234*
TCI-R: Cooperativeness		-.047	.037	-.097*	-.091*	-.107*	.001
TCI-R: Self-Transcendence		.013	.119*	.184*	.193*	.213*	.105*
Gambling type (strategic)		.087*	.064*	.059*	.057*	.038	.051*
	*Block summary: ΔR* ^*2*^	***.007*** ^***✝***^	***.004*** ^***✝***^	***.003*** ^***✝***^	***.003*** ^***✝***^	*.001*	***.002*** ^***✝***^

Beta-coefficients (β) in multiple regressions. ΔR^2^: increase in R^2^. *Bold: significant Beta-coefficient. ^✝^Bold: significant R^2^.

TCI-R: Temperament and Character Inventory-Revised.

SOGS: South Oaks Gambling Screen.

SCL-90-R: Symptom Check List- 90-Revised.

## Discussion

The aim of the present study was to assess clinical and psychopathological differences and personality profiles between two groups of pathological gamblers who were divided according to their gambling preferences. Specifically, we compared patients with GD who chose strategic gambling where individual skills are emphasized (cards, poker, craps, sports betting, or stock market investment) with patients with GD who chose non-strategic gaming which relies on chance (slot machines and lottery).

Results showed that the SG group was mostly comprised of males, with an earlier onset of the disorder and a higher level of academic achievement. These findings are in agreement with other research studies [36,37], which show that SG are usually young males of an average age of 24 years [38]. It can be hypothesized that these types of games attract young men due to the high levels of competitiveness found in this population [39]. The lack of females in the SG group can also be explained by the link between these gambling activities and risk taking behavior, which is known to be less common among females [40]. The higher number of women in the non-SG group is in accordance with other studies [41] which have described the possible role of this type of gambling as an emotional regulator. It is hypothesized that the motivation that drives women to gamble is related to the avoidance of negative emotions and as a way to help them to cope with high levels of stress. The role of non-SG in mood regulation can also explain the high co-morbidity found between non-SG and tobacco addiction [42,43], which is further supported by the results of this study. Gambling behavior, as well as smoking, plays a role in dealing with negative emotions and stress [44]. Moreover, these results confirm the previously described association between non-SG and the regulation of negative emotional states [9,13,16].

The study also found that individuals with SG tend to engage in more than one specific type of problematic game and to bet higher amounts of money, which may indicate greater severity which has also been found in previous studies [23]. In order to attempt to explore the issue of severity further, this study also analyzed the psychopathology comorbid in this population and found that SG presented with higher levels of psychopathology than non-SG. Symptoms such as excessive rumination (planning and thinking of past gambling episodes), depression and anxiety, as well as hostility and isolation appear to be more frequent in the SG group. Previous studies [10] have already suggested that SG was associated with affective disorders, which are considered a risk factor for gambling, or are secondary to the gambling problem itself [45]. Gamblers progressively become cut off from their family and social life which maintains their psychopathology. As a consequence of this, a vicious cycle develops where lack of social or interpersonal contact or support and depressed mood are at the centre of the gambling activity. Unfortunately, the cross-sectional nature of this study does not allow us to determine whether if, in the case of non-strategic gamblers, poor affective state precedes the GD (with gambling behavior a maladaptive mechanism of emotion regulation) as several authors have suggested to be the case for females [46,47], while for strategic gamblers, emotional disturbances result from the consequences of GD [48].

Future studies may want to investigate whether depression is a mechanism to deal with negative emotions arising from gambling, or is a predisposing factor of this behavior.

The study also investigated in depth the personality characteristics of the two groups of patients with GD, and found that SG had higher levels of novelty-seeking and lower levels of cooperation. Cooperation is a trait which is believed to be a socially protective factor. The low levels of cooperation found among the SG indicate greater global dysfunction in this group of GD and these findings are in agreement with previous research [10,11].

The final aim of this study was to investigate whether specific gambling preferences could predict the severity of the disorder. The study found that from a socio-demographic point of view, there was an association between gender (male) and psychopathology (lower levels). Several studies show greater emotional instability and psychopathology in women, especially with regard to depression and anxiety [47,49]. However, our results do not support this association with regard to the hostility trait and gender. Previous studies have identified that women with addictive behavior show high levels of hostility, when compared with women from the general population [50] and show no differences in the levels experienced by males [51]. Furthermore, this study showed an association between an older age and a higher severity of the disorder. In this line, several studies have reported that the most severe consequences of gambling are observed among middle-aged individuals. Often, young people do not have an economic situation that allows them to invest a significant amount of money in gambling. Therefore, the detrimental effects of the GD, such as the accumulation of debts, bankruptcy or its interference in family or work life, are usually primarily associated with older patients [52,53]. Moreover, an older age has been associated with elevated depression and PST scores (an index of the SCL-90-R that measures the amplitude and severity of the psychopathology). This result is not surprising given that, as individuals become older, they are more exposed to stressful life situations such as economic and family responsibilities, health problems, etc. This suggests that old people may use gambling as a regulatory mechanism of negative emotions [54].

High scores on Novelty Seeking, Harm Avoidance, Persistence, and Self-Transcendence were found to be predictors of higher psychopathology and greater severity of the disorder. Specifically, high scores on Self-directedness were significantly predictive of low levels of both psychopathology and severity of the gambling problem. Self-directness is the ability to regulate and adapt behavior to the demands of a situation in order to achieve personally chosen goals and values. Self-directedness is conceptually related to locus of control [55] and research has found that low self-directedness is a major common feature of personality disorders and it has been associated with different psychiatric disorders with risk for substance abuse [56], eating disorders [57], and other mental problems [58].

The results of this study also indicated that the high level in TCI-R Reward Dependence and Cooperativeness were not found to have an effect on the severity of the disorder, but predicted lower levels of psychopathology and a reduced intensity of somatic and psychic suffering (measured with the GSI, PST and PSDI indices), which suggests that it may be an effective protective factor. These traits reflect social tolerance, empathy, altruism, and reconciliation capabilities, which may indicate better social support, which is known to affect psychopathology and severity. In this sense, there are also many studies that consider sociability as a protective factor for various disorders [59]. However, consistent with the literature, the most characteristic feature of GD is novelty-seeking. Elevated levels on this scale are generally obtained by comparing GD with controls, demonstrating elevation in both games (chance *versus* skills) [60]. In our study, when comparing the two groups of GD, it is noted that the SG obtained greater novelty-seeking, results that are in accordance with those obtained by Bonnaire [15]. In addition, this group had greater severity of the disorder and psychopathology, a finding that is consistent with multiple studies [61].

Although the strength of the study is the large clinical sample analyzed, there are several features of the sample that need to be noted. Specifically, there were more men than women and a greater number of slot machine gamblers compared to strategic types, meaning that both women and strategic gamblers are less well represented. In addition, all data was collected at a single facility and from individuals who were voluntarily attending services for gambling disorder and so may not be representative of all gamblers, rather those more motivated to be helped. While the groups did differ significantly in their sociodemographic and psychopathological status, and in personality variables, the effect sizes were small. Therefore, although both groups present some distinctive clinical features, these differences are not as large as expected. Finally, the cross-sectional nature of the data does not allow for direction of effects for to be determined. Therefore, caution should be used when translating the results to the general population of gamblers.

## Conclusion

In conclusion, this study found that strategic or skill-based gamblers suffer from higher level of psychopathology and present with a different personality profile than chance-based or non-strategic gamblers. In addition, non-strategic gambling appears to be linked with a dysfunction in the ability to regulate negative emotions. The clinical significance of these finding is in relation to the management of these conditions, as patients may respond to different interventions depending on the different roles that the gambling activity may have in their lives. The role of emotional regulation and depression in the maintaining mechanism of GD and the protective factor of sociability and social support may indicate that specific therapies, such as interpersonal psychotherapy, could be adapted to treat these patients. Interpersonal Psychotherapy has been used for the treatment of depression and has been adapted to be used in different groups of patients, such as depressed adolescents [62], social phobia [63], bipolar disorder [64], the elderly [65] or dysthymic disorder [66], between others. It aims at targeting interpersonal problems that maintain psychopathology [67]. Future studies investigating the role of interpersonal problems in the development and maintenance of GD may help inform the development of treatments for these conditions. Also, clinically, these results are important, not only in relation to the need to design therapies tailored to the specific psychopathological and personality characteristics of the patient, but also in relation to prevention. From this perspective, these findings may be useful in informing campaigns in the media about best practices in gambling activity, risks associated with certain types of gambling and/or personality profiles and advertising regulation.

## Abbreviations

GD: Gambling disorder
DSM: Diagnostic and statistical manual of mental disorders
SG: Strategic gamblers
Non-SG: Non-strategic gamblers
SOGS: South Oaks gambling screen
SCL-90-R: Symptom Check List- 90-Revised
GSI: Global severity index
PST: Positive symptom total
PSDI: Index positive symptom distress
TCI-R: Temperament and character inventory-revised
CEIC: Ethics Committee
CBT: Cognitive-behavioral therapy
ANOVA: Analysis of variance.
